# *Parabacteroides distasonis* alleviates enterotoxigenic *Escherichia coli*-induced diarrhea in mice by mediating gut microbiota

**DOI:** 10.3389/fmicb.2025.1716958

**Published:** 2026-02-06

**Authors:** Hongliang Chen, Jinshuo Gong, Jia Wang, Yang Li, Wenjie Yang, Jianan Guo, Jie Wang, Yongxiang Wang, Lu Zhou, Lei Zhang, Chunwei Shi, Di Zhang

**Affiliations:** 1 College of Veterinary Medicine, Jilin Agricultural University, Changchun, China; 2 Jilin Provincial Key Laboratory of Animal Microecology and Healthy Breeding, Jilin Provincial Engineering Research Center of Animal Probiotics, Jilin Agricultural University, Changchun, China; 3 Engineering Research Center of Microecological Vaccines (Drugs) for Major Animal Diseases, Ministry of Education, Jilin Agricultural University, Changchun, China; 4 Jilin Agricultural University, Changchun, China

**Keywords:** *P. distasonis*, enterotoxigenic *Escherichia coli*, gut microbiota, proinflammatory cytokines, tight junction proteins

## Abstract

Enterotoxigenic *Escherichia coli* (ETEC) is among the major pathogens responsible for diarrhea in young piglets, leading to significant economic losses. Elucidating the interaction between gut microbiota and ETEC is crucial for developing alternative therapeutics that do not rely on antibiotics. This study aimed to investigate the mitigative effects of *Parabacteroides distasonis* on ETEC-induced diarrhea in a mouse model. Twenty-four SPF female BABL/c mice were equally allocated into four treatment groups: sham challenge (CON), Escherichia coli K88 challenge (ETEC), *P. distasonis* treatment with ETEC K88 challenge (PD), and *E. coli* Nissle 1917 treatment with ETEC K88 challenge (ECN) groups. The experiment lasted for 14 days, spinning from 8 days before to 6 days after the first ETEC K88 challenge (day 9). The mice in the ETEC, PD and ECN groups were gavaged with ETEC for 5 consecutive days with a dosage of 2 × 109 CFU twice per day, whereas the PD and ECN groups were inoculated with 2 × 108 CFU *P. distasonis* and *E. coli* Nissle 1917 once per day, respectively. Both reverse transcription PCR and western blotting revealed significantly increased levels of tight junction proteins (ZO-1, occludin and claudin-1) and ELISA exhibited significantly decreased TNF-α, IL-1β and IL-8 levels. Histopathological analysis revealed that *P. distasonis* repaired intestinal damage and restored villus length caused by ETEC infection. Furthermore, 16S RNA sequencing indicated that gut microbiota dysbiosis was ameliorated by enhancing the abundance of beneficial bacteria the Lachnospiraceae_NK4A136_group and *B. uniformis*. Overall, our study suggested that *P. distasonis* could alleviate ETEC-induced diarrhea by restoring intestinal barrier integrity, and regulating gut microbiota and proinflammatory cytokines, which serves as an alternative strategy to control ETEC infection.

## Introduction

Enterotoxigenic *Escherichia coli* is the main pathogen causing fatal diarrhea in children under 5 years and piglets during neonatal and postweaning period (Fleckenstein et al., 2010; Gresse et al., 2017). Host specific colonization fimbriae such as K88 (F4), K99 (F5), F41, 987P (F6), and F18, significantly contribute to the virulence of porcine ETEC, by facilitating the adhesion of the bacteria to the intestinal epithelium, which is crucial for the subsequent delivery of enterotoxins including heat-stable enterotoxin and heat-labile enterotoxin (Duan et al., 2012; von Mentzer and Svennerholm, 2024). ETEC K88 infection is the most prevalent in the global pig industry, leading to considerable economic losses (Nagy and Fekete, 2005; Luppi et al., 2016). Antibiotic growth promoters (AGPs) have been extensively used for decades to prevent *E. coli* and other bacterial pathogen. However, with the emergence of antibiotic resistance and drug residues, the European Union and several Asian countries have prohibited the use of AGPs in animal production (Chatterjee and Paul, 2019; Miller and Arias, 2024). Consequently, there is an urgent need for alternative strategies to prevent porcine ETEC infection.

Probiotics are viable, beneficial microorganisms that contribute to host health and intestinal homeostasis (Sarita et al., 2024). A large body of research has focused on probiotic strains of *Lactobacillus, Bacillus*, and *Bifidobacterium*, which exert protective roles against ETEC infections (Jiang et al., 2024). *L. zeae* LB2 recovered microbial alpha diversity and affected functional potentials of gut microbiota to alleviated ETEC-induced diarrhea in piglets (Zhang et al., 2022). *Bacillus halotolerans* SW207 upregulated tight junction proteins and suppressed the activation of the TLR4/MyD88/NF-κB pathway to reduce inflammation in ETEC infected weaned piglets (Li et al., 2024). *Bifidobacterium bifidum* FL228.1 and FL276.1 enhanced intestinal IgA levels and modulate epithelial barrier function to combat ETEC infection (Zhao et al., 2025). *P. distasonis* is a re-classified commensal bacterium in 2006 with a former name *Bacteroides distasonis*, which colonizes in the gastrointestinal tract of humans and animals (Ezeji et al., 2021). Growing evidence suggests that *P. distasonis* could modulate various diseases, including inflammatory bowel disease, obesity, colorectal cancer, bladder cancer, multiple sclerosis, type II diabetes, testicular dysfunction, rheumatoid arthritis, acute ischemic stroke, and hepatic fibrosis (Ezeji et al., 2021; Cekanaviciute et al., 2017; Del Chierico et al., 2017; Verdam et al., 2013; Wang et al., 2019; Han et al., 2021; Sun et al., 2023; Zhao et al., 2023; Wei et al., 2024; Wang B. et al., 2024). The potential application of *P. distasonis* as a strategic alternative to control porcine ETEC infections is of significant interest.

The pathogenicity of ETEC infection has been extensively investigated utilizing intestinal porcine epithelial cells-jejunum 2 (IPEC-J2) *in vitro* over the last decade, with a focus on pathogen adhesion, barrier function, and proinflammatory cytokines. Tight junction proteins (TJs) including occludin, claudins, and zonula occludens (ZO), are significant components of intestinal barriers against pathogens. ETEC-induced barrier dysfunction manifesting as reducing levels of TJs results in diarrhea in weaned piglets (Xu et al., 2021). Recently, an increasing number of studies have been conducted *in* vivo, emphasizing the dissection of the gut microbiota during ETEC infection using *16S* rRNA sequencing. Zhang et al. reported a decreased abundance of *Prevotella, Ruminococcaceae, Lactobacillus, Alloprevotella*, and *Flavobacterium* in ETEC infected piglets (Zhang et al., 2022). Wang et al. found that ETEC infection significantly increased the abundance of *Escherichia* and *Streptococcus*, while decreased the abundance of *Gemmiger, Lactiplantibacillus, Enterococcus, Levilactobacillus, Weissella, Clostridium, Parabacteroides, Phascolarctobacterium*, and *Collinsella* in weaned piglets (Wang B. et al., 2024). The aim of the current study is to determine the protective role of *P. distasonis* on ETEC K88 infection, focusing on the alterations in gut microbial populations as revealed by *16S* rRNA gene sequencing.

## Materials and methods

### Bacterial strains and cells

*P. distasonis* was purchased from American Type Culture Collection (ATCC 8503) and cultured in brain-heart infusion broth under anaerobic environment at 37 °C. *E. coli* strains Nissle 1917 and ETEC K88 was maintained in our laboratory as a gift from Prof. Guoqiang Zhu, Yangzhou University, China (Li et al., 2024), and incubated in Luria-Bertan broth. All the strain cells were harvested by centrifugation at 2,000 × g for 10 min at 4 °C after incubation. Then the pellets were washed with sterile phosphate buffer saline (PBS) twice and diluted for further assays.

IPEC-J2 cells were cultured in Dulbecco's Modified Eagle medium/F12 medium with 10% FBS and 1% antibiotics (100 μg/mL streptomycin and 100 U/mL penicillin) at 95% relative humidity, 37 °C under an atmosphere containing 5% CO_2_.

### Animals and experimental design

The specific pathogen free (SPF) female BABL/c mice (4–5 weeks old) were provided by Beijing Huafukang Bioscience Co., Ltd, China. Mice were housed under constant temperature and humidity, with a 12 h light–dark cycle and access to food and water *ad libitum*. The animal experimental protocol was approved by the Animal Management and Ethics Committee of Jilin Agricultural University (Ethics Approval No. 2023-1210-001).

Animals with similar body weights were assigned into four groups (six mice per group). The four treatment groups were described as follows. (1) negative control group (CON): without ETEC K88 challenge. (2) ETEC K88 challenge group (ETEC). (3) *P. distasonis* treatment + ETEC K88 challenge group (PD). (4) *E. coli* Nissle 1917 treatment + ETEC K88 challenge group (ECN). The whole experiment lasted for 14 days. On day 1–5, mice in group CON and ETEC were gavaged with 200 μL of protectant of *Saccharomyces boulardii*. Meantime, mice in PD or ECN group were gavaged with 200 μL of *P. distasonis* or *E. coli* Nissle 1917 suspension (1 × 10^9^ CFU/mL; 1 × 10^9^ CFU/mL), respectively. On day 6–8, all animals in each group were administrated streptomycin (5 g/L) to jeopardize the gut microbiota. Then on day 9–13, except CON group, all other groups were gavaged with ETEC K88 twice a day both in the morning and the evening. Moreover, mice in PD group were intragastrically administrated with 200 μL of *P. distasonis* (1 × 10^9^ CFU/mL), and mice in ECN group were administrated of the equivalent volume of *E. coli* Nissle 1917. Mice in CON group were given 200 μL of PBS.

The body weights were recorded on day 8–14. At the same time, diarrhea assessment was performed. The severity of ETEC-induced diarrhea in mice was scored as the following standard (Zhang et al., 2017); 0: normal stool; 1 = soft stool; 2 = soft and slightly wet stool; 3 = wet, unformed stool with moderate perianal staining; 4 = watery stool with severe perianal staining. On day 8 and 14, the stool samples of each group were collected and stored at −80 °C for further sequencing analysis. In addition, on day 14, blood samples were collected from the orbital venous plexus and centrifuged to get the serum and the intestinal tissues were also excised.

### ETEC K88-infected IPEC-J2 cells model

IPEC-J2 cells were seeded in 96 well plates (5 × 10^4^ cells/well), then the cells were incubated with ETEC K88 alone, ETEC K88 with *P. distasonis*, ETEC K88 with *E. coli* Nissle 1917, respectively. After incubation for 1, 3, and 6 h, all the supernatants were collected for further ELISA and RT-PCR assays. The effects of ETEC K88, *P. distasonis* and *E. coli* Nissle 1917 on the viability of IPEC-J2 cells were evaluated utilizing cell counting kit-8 (CCK-8, Apexbio Technology LLC, USA), and multiplicity of infection (MOI, MOI = 10:1 or MOI = 100:1) of bacteria were detected. Experiments were performed in triplicates.

### Histomorphology analysis

Jejunal and ileal tissues were removed, fixed in 4% paraformaldehyde and embedded in paraffin wax. Then tissues were dehydrated, sliced, stained with hematoxylin and eosin (H&E), finally examined by light microscope (Leica Microsystems, Wetzlar, Germany). Villus lengths of the tissues were also measured.

### Enzyme-linked immunosorbent assay

The levels of IL-8, TNF-α, and C-reactive protein (CRP) in serum, the concentrations of TNF-α, IL-1β, and IL-8 in supernatants of IPEC-J2 cells after ETEC challenge were determined utilizing commercially available ELISA kits (Enzyme-Linked Biotechnology Co., Ltd, Shanghai, China) for murine IL-8, TNF-α, CRP and for porcine TNF-α, IL-1β, and IL-8. According to the manufacturer's instructions, all assays were performed in triplicates.

### RT-PCR

Total RNA from jejunal and ileal tissues was isolated and reverse transcribed into complementary DNA. RT-PCR procedure was carried out to determine the mRNA abundance of ZO-1, Occludin, Claudin-1, TNF-α, IL-8, and CRP utilizing Applied Biosystems 7500 PCR instrument (Life Technologies, Carlsbad, CA, United States). The 2^−ΔΔCt^ method was performed for calculating the mRNA levels and normalized with reference gene (β-actin). The PCR conditions were 95 °C for 2 min, followed by 40 amplification cycles (denaturation at 95 °C for 15 s, annealing at 60 °C for 30 s, and extension at 60 °C for 20 s). All analyses were carried out in triplicates. The primer sequences for target genes were synthesized by Sangon Biotechnology (Shanghai, China) and listed in Supplementary Tables 1, 2.

### Immunohistochemistry

The ileum sections were blocked with 5% goat serum and 0.3% Triton X-100 in PBS for 1 h at room temperature. Then the blocking solution was discarded and the sections were stained with primary antibodies (occludin, claudin-1, and ZO-1) at 4 °C overnight. Further, the sections were washed and treated with goat anti-rabbit IgG secondary antibody. At last, diaminobenzidine was added to sections and examined under fluorescence microscopy (DMIL LED, Leica Microsystems, Germany).

### *16S* rRNA sequencing, bioinformatics and sequencing data analysis

The *16S* rRNA gene sequencing analysis was following the protocol used in our laboratory (Chen et al., 2021). The V3–V4 hypervariable region of the *16S* rRNA gene was used in this study (Nobar_341F, CCTACGGGNGGCWGCAG; Nobar_805R, GACTACHVGGGTATCTAATCC). The values of Chao 1, ACE, and Shannon-Wiener indices were calculated to evaluate the alpha diversity of the samples. Principal coordinates analysis (PCoA) of the weighted UniFrac metrics was used to visualize differences in microbial community structure. Linear discriminant analysis (LDA) coupled with the effect size (LEfSe) algorithm was conducted to identify the significant microbial differences among all the groups. The raw reads were deposited into the NCBI Sequence Read Archive database (SRA accession number SRP626360, BiopProject accession number PRJNA1333859).

### Statistical analyses

The data were analyzed and visualized by GraphPad Prism 8 (GraphPad Software Inc., CA, USA). One-way ANOVAs were utilized to analyze the differences between different groups in ELISA assays and RT-PCR detections. All experiment data were expressed as the mean ± standard error (SE). *P*-values of < 0.05 were considered significant. Statistical significance is indicated as ^*^P < 0.05, ^**^P < 0.01, ^***^P < 0.001 and ^****^P < 0.0001.

## Results

### Establishing the murine ETEC K88-induced diarrhea model

**Figure 1:**
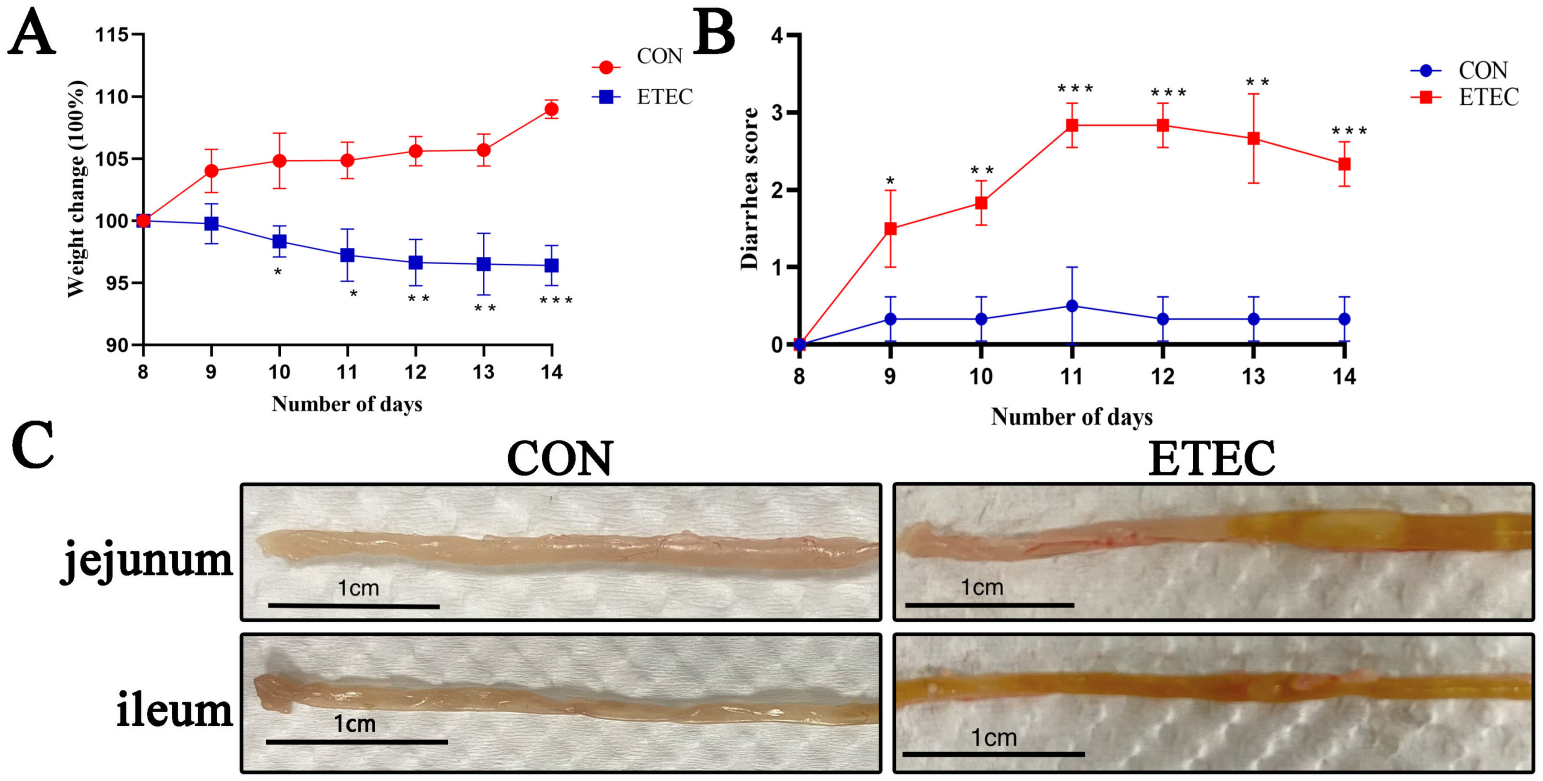
Effects of ETEC K88 infection on body weight changes in BALB/c mice **(A)**. ETEC K88-induced diarrhea score in mice **(B)**. Gross images of the intestinal tissues in ETEC K88 infected mice **(C)**. All of the data are expressed as the mean ± standard error (SE). Statistical significance is indicated as ^*^P < 0.05, ^**^P < 0.01, and ^***^P < 0.001.

ETEC K88-induced diarrhea model in BALB/C mice successfully established, following the protocol in previous research (Yue et al., 2020). Body weight and diarrhea-scores were evaluated daily from day 8 to day 14. The body weights of BALB/C mice were significantly decreased on day 10, 11, 12, 13, and 14, compared with control group (Figure 1A). Meantime, significant increased diarrhea scores in model groups were observed since day 9 to day 14 (Figure 1B). As shown in Figure 1C, hemorrhage in jejunal and ileal tissues of model groups were also observed.

### *P. distasonis* alleviates ETEC K88-induced diarrhea *in vivo*

**Figure 2:**
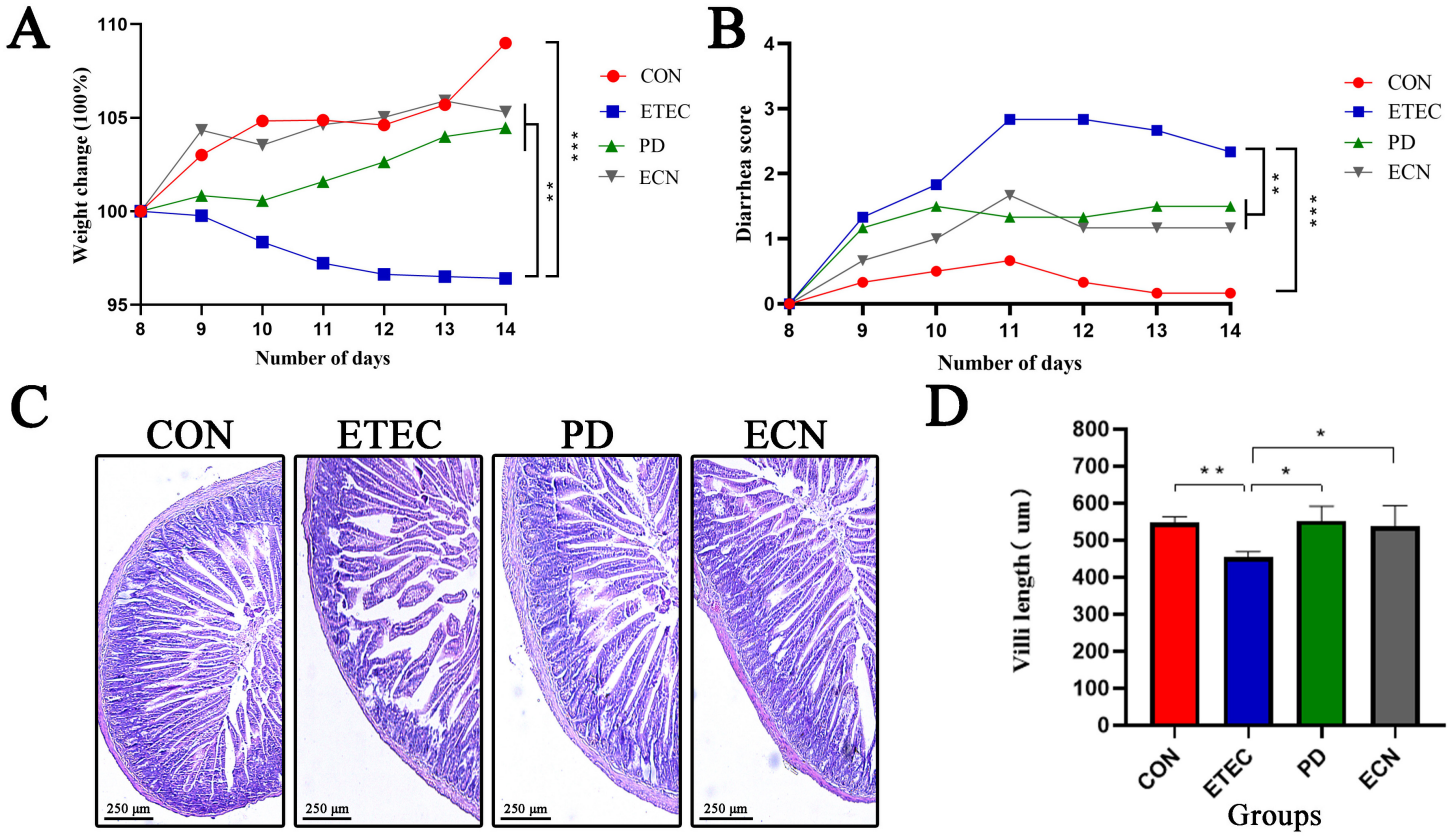
Effects of *P. distasonis* and *E. coli* Nissle 1917 treatment on body weight changes in ETEC K88 infected BALB/c mice **(A)**, on diarrhea score in infected BALB/c mice **(B)**. Jejunal morphology **(C)** and villi length of jejunum in each group **(D)**. All of the data are expressed as the mean ± standard error (SE). Statistical significance is indicated as ^*^P < 0.05, ^**^P < 0.01, and ^***^P < 0.001.

The body weights were monitored during the ETEC K88 challenge. Body weight of BALB/C mice in ETEC group decreased by 3.6% during the challenge period. While the weight of mice in CON group, PD group, and ECN group increased by 8.7%, 4.5%, and 5.3%, respectively, exerting significant differences compared with the ETEC group (Figure 2A). Moreover, Figure 2B indicated that on day 14, the diarrhea score of BALB/C mice for each group were as follows: ETEC group (2.3), CON group (0.2), PD group (1.3). ECN group (1.2). As shown in Figures 2C, D, the villi length of jejunum in BALB/C mice was measured. ETEC K88 significantly shortened the villi length by 17.2% (*P* < 0.01). What's more, in comparison with ETEC group, the length of villi both significantly increased in PD and ECN group (*P* < 0.05; *P* < 0.05).

**Figure 3:**
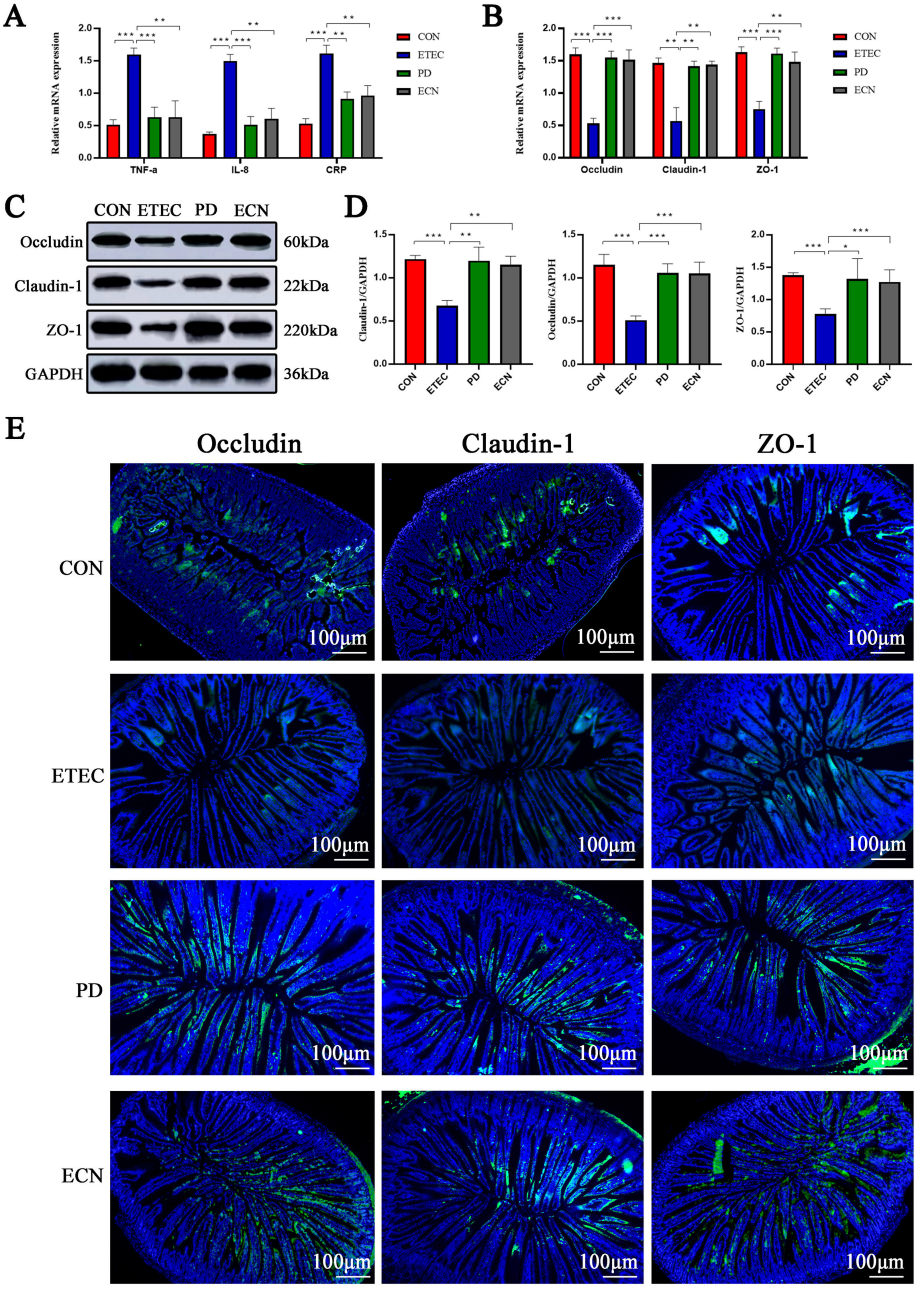
Effects of *P. distasonis* and *E. coli* Nissle 1917 treatment on mRNA expression levels of cytokines **(A)**, tight junction proteins **(B)** in intestinal tissues. WB assay results for tight junction proteins **(C, D)**. Immunohistochemistry results of tight junction proteins in intestinal tissues **(E)**. All of the data are expressed as the mean ± standard error (SE). Statistical significance is indicated as ^*^P < 0.05, ^**^P < 0.01, and ^***^P < 0.001.

To elucidate the protective effects of *P. distasonis* on ETEC K88-challenged mice, inflammatory cytokine and tight junction protein levels in the jejunum tissues were detected by RT-PCR. ETEC significantly increased TNF-α, IL-8, and CRP mRNA levels than those of the control group. Comparing with ETEC group, NF-α, IL-8, and CRP mRNA levels were significantly reduced in PD and ECN groups, respectively (Figure 3A).

Moreover, we found that a significant reduction in the mRNA expression of occludin, claudin-1 and ZO-1 in the ETEC group, comparing with the CON group, respectively. The mRNA expression levels of occludin, claudin-1 and ZO-1 were significantly higher in PD or ECN group than those in ETEC group (Figure 3B). WB assay was also conducted to detect the protein expression levels and exhibited a similar result with the RT-PCR (Figures 3C, D).

Immunohistochemistry was also performed to evaluate the protein expression levels and distributions of occludin, claudin-1 and ZO-1, the staining indicated that the proteins localized onto the villi and levels were significantly reduced in ETEC group in relation to CON group. Moreover, the protein expression levels were significantly higher in PD or ECN group than in ETEC group (Figure 3E).

### *P. distasonis* protected ETEC K88-induced IPEC-J2 injury *in vitro*

**Figure 4:**
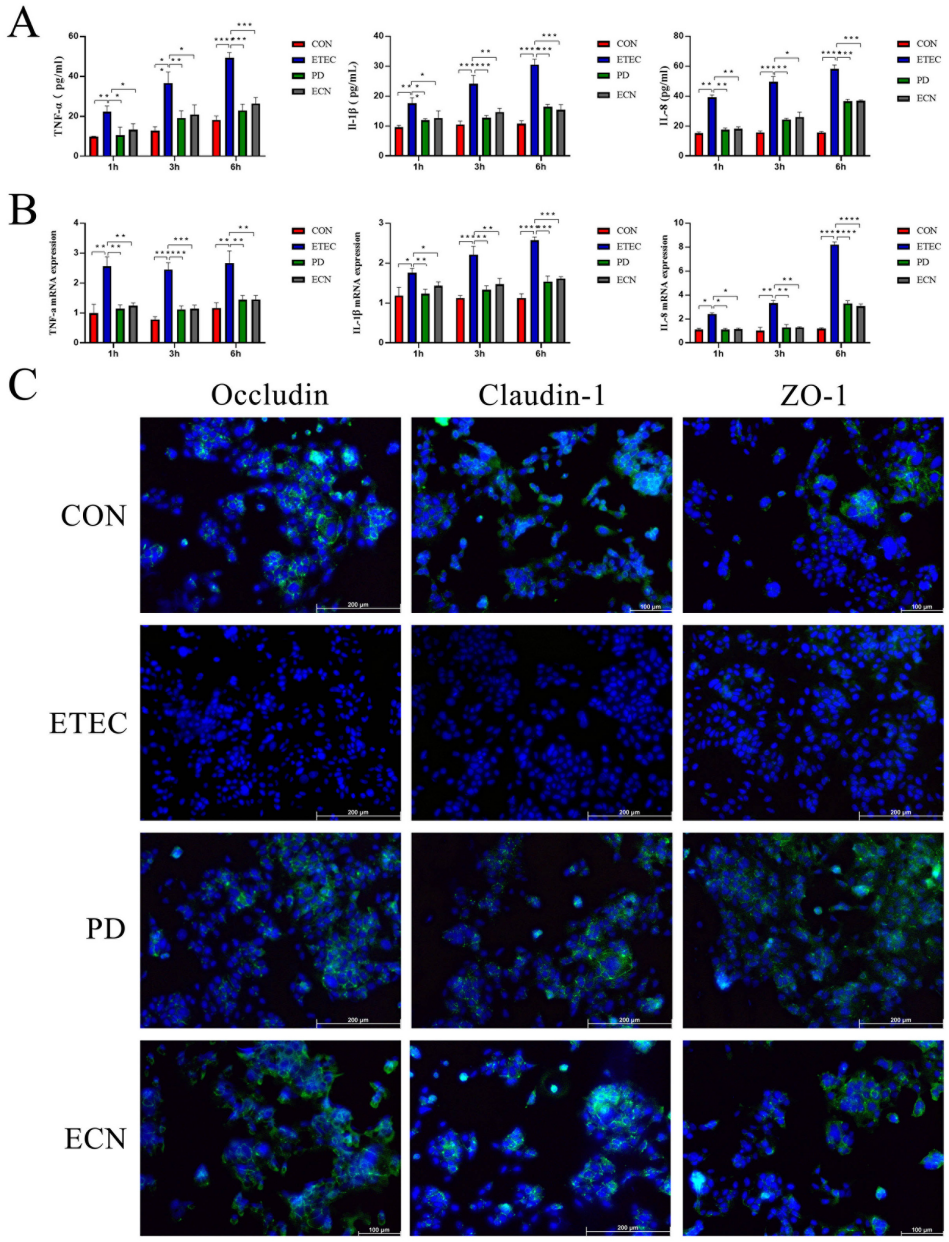
ELISA results of proinflammatory cytokines **(A)** and relative mRNA expressions of proinflammatory cytokines **(B)**. Immunohistochemistry results of tight junction proteins in IPEC-J2 cells **(C)**. All of the data are expressed as the mean ± standard error (SE). Statistical significance is indicated as ^*^P < 0.05, ^**^P < 0.01, and ^***^P < 0.001.

Under the conditions of MOI = 10 or MOI = 100, both *P. distasonis* and *E. coli* Nissle 1917 exhibited no cytotoxicity on IPEC-J2 cells, as shown in Supplementary Figure 1. Then to explore the immunomodulatory effects of *P. distasonis* and *E. coli* Nissle 1917 on the inflammatory processes of ETEC K88-induced IPEC-J2 injury, we analyzed the gene expression levels of cytokines TNF-α, IL-1β, and IL-8. At incubation time of 1, 3, and 6 h, all cytokines were significantly increased in ETEC group than CON group. While the mRNA expression levels of cytokines were all significantly down-regulated in PD or ECN group than those in ETEC group. At the protein level, ELISA assay also confirmed a similar result with the qPCR result (Figures 4A, B). Immunohistochemistry results showed that a uniform distribution of tight junction proteins in CON group. However, tight junction disruption was observed in ETEC group and relatively smaller disruptions of tight junctions were observed in PD and ECN group (Figure 4C).

### *P. distasonis* modulated gut microbiota in mice model infected by ETEC K88

Twenty-four samples from groups of CON8, ETEC8, PD8, ECN8, CON14, ETEC14, PD14, and ECN14, produced a total of 2,258,592 sequences after assembly and filtration, and the average sequence length was 417.5 bp (Supplementary Figure 2).

**Figure 5:**
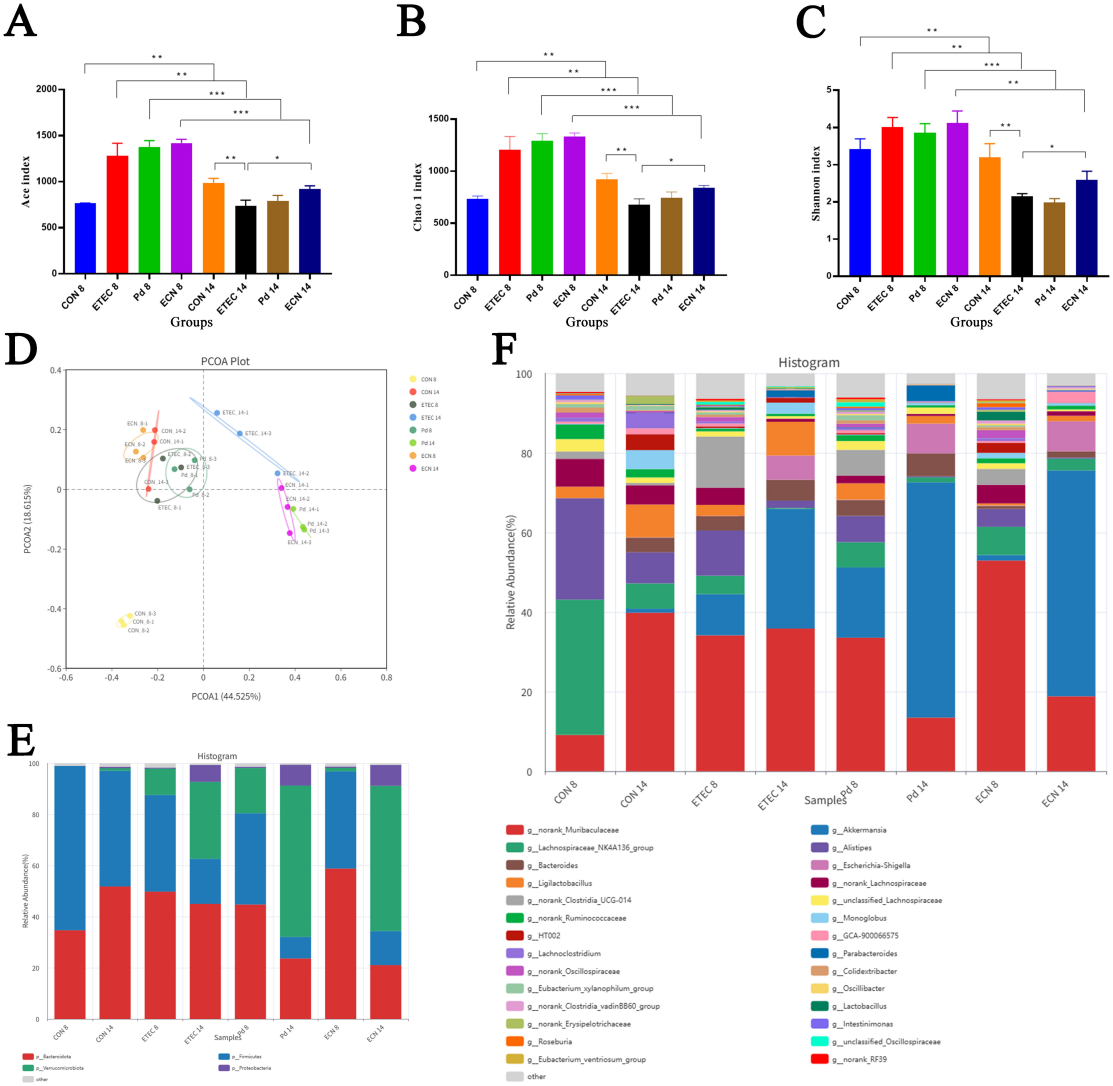
Alpha diversity indices including ACE index **(A)**, Chao 1 index **(B)** and Shannon index **(C)** in each group. Principal component analysis of the structure of the gut microbiota **(D)**. The relative abundances of the gut microbiota at the phylum **(E)** and genus **(F)** levels. All of the data are expressed as the mean ± standard error (SE). Statistical significance is indicated as ^*^P < 0.05, ^**^P < 0.01, and ^***^P < 0.001.

The Chao 1 and ACE indices, which measure microbial richness, the Shannon index, which estimate microbial species biodiversity, were computed to evaluate the gut microbiota alpha diversity. Chao 1, ACE, and Shannon indices all showed significant lower levels in ETEC14, PD14, and ECN14 group compared with the corresponding ETEC7, PD7, and ECN7 group, respectively, indicating decreased alpha diversity over time after ETEC K88 infection. What's more, these three indices also showed significant lower abundance in ETEC 14 group than in CON14 group. However, these indices showed significant higher abundance in ECN14 group than in ETEC14 group, and higher abundance in PD14 group than ETEC14 group, separately. Our data revealed that both *P. distasonis* and *E. coli* Nissle 1917 could recover the microbial richness and species biodiversity in mice after ETEC K88 infection, especially *E. coli* Nissle 1917 (Figures 5A–C).

PCoA (Principal Coordinates Analysis) analysis showed that there was a large alteration in the gut microbial structure in the ETEC K88 infected group. There was little variation between PD14 group and ECN14 group, indicating a similar intestinal microbe structure. In conclusion, the impact of *P. distasonis* and *E. coli* Nissle 1917 on ETEC K88 infected microbial community structure were in agreement with the alpha diversity results (Figure 5D).

At the phylum level, Firmicutes, Bacteroides, Proteobacteria, and Verrucomicrobia dominated the gut microbiota in all groups. At day14, compared with CON14 group, the ETEC14 group had lower abundance of Bacteroides, significant lower abundance of Firmicutes (*P* < 0.01), higher abundance of Proteobacteria and significant higher abundance of Verrucomicrobia (*P* < 0.0001) (Figure 5E).

**Figure 6:**
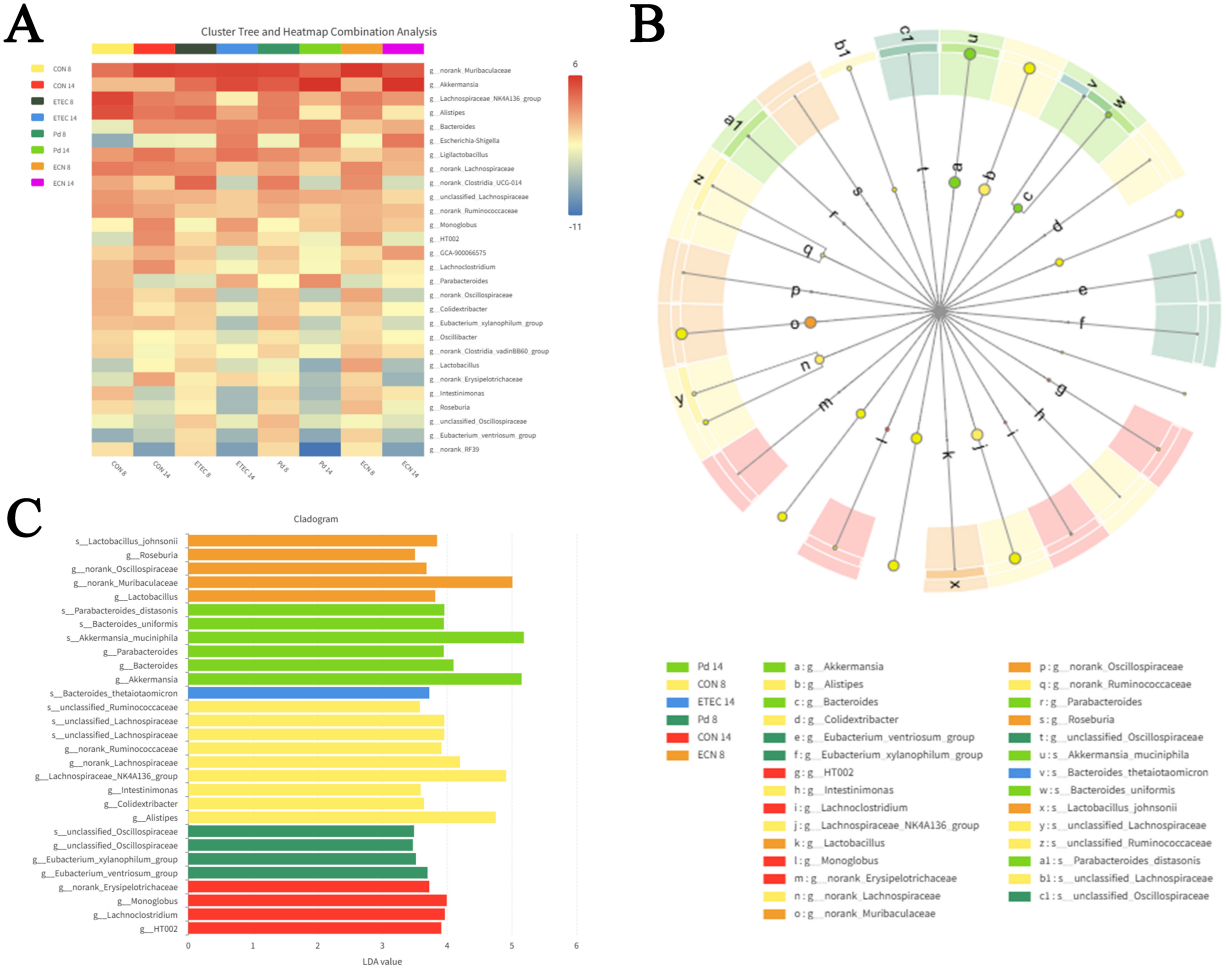
Heatmaps plot depicting the relative abundance of intestinal microbial genus **(A)**. Cladogram of the LEfSe analysis **(B)** and LDA score obtained from the LEfSe analysis **(C)**.

At the genus level, the no rank Muribaculaceae, *Akkermansia*, the Lachnospiraceae_NK4A136_group, *Alistipes, Bacteroides, Escherichia*-*Shigella, Ligilactobacillus*, the no rank Lachnospiraceae, the no rank Ruminococcaceae and *Monoglobus* were the most prevalent (Figure 5F). In addition, the 30 most abundant bacteria genera for each group were shown in Figure 6A.

To identify the significant differences in the population of microbes in ETEC K88 infected mice, a LEfSe analysis was also conducted. The taxonomic cladogram and LDA score diagram were computed to display the effects of ETEC K88 infection on gut microbiota (Figures 6B, C). The abundance of *Bacteroides thetaiotaomicron* was significantly increased in ETEC14 group. The abundance of *P. distasonis, Bacteroides uniformis, Parabacteroids, Bacteroides, Akkermansia muciniphila* were all significantly increased in PD14 group.

## Discussion

Probiotics have the potential to take place of antibiotics to prevent ETEC-induced diarrhea. A growing number of research has demonstrated that *Lactobacillus* and *Bifidobacterium* bacteria could combat ETEC infection *in vitro* and *in vivo* (Zhang et al., 2022; Roselli et al., 2006; Wang et al., 2018). However, there are few studies investigating the effects of *P. distasonis* on ETEC K88*-*induced diarrhea. In this study, we examined the impact of *P. distasonis* on gut microbial community and inflammatory cytokine expression in the intestinal tissue of BALB/c mice infected by ETEC K88. We found that supplementation with *P. distasonis* could recover the gut microbial diversity, reduce pro-inflammatory cytokines and modulate ileal barrier function, leading to a significant alleviation of diarrhea symptoms.

It's acknowledged that a stable ETEC K88-infected mice model was hard to accomplish (Dubreuil, 2017). In our study, we adhered to the protocol outlined by Yue et al. (2020) and Zhang et al. (2025) and successfully utilized BALB/c mice to establish the ETEC K88-infected model. The success of the model was evidenced by parameters such as decreased body weight, elevated diarrhea scores, and damaged intestinal morphology, consistent with previous research (Wang et al., 2018; Wang Y. et al., 2024; Tang et al., 2019). The addition of *P. distasonis* and *E. coli* Nissle 1917 alleviated these symptoms. Furthermore, treatment with *P. distasonis* and *E. coli* Nissle 1917 resulted in significantly increased villi lengths, indicating their immune protective roles, as villi are crucial for enteric absorption and secretion. Then we elucidated the protective effects of these two novel probiotics both *in vivo* and *in vitro*.

ETEC K88 infection promoted the gene expression of TNF-α, IL-8, and CRP cytokines in ileum tissue. Additionally, *P. distasonis* and *E. coli* Nissle 1917 both exhibited beneficial impact in down-regulating cytokines of TNF-α, IL-8, and CRP caused by ETEC K88, and ability to down-regulate pro-inflammatory cytokines for *P. distasonis* was a little bit stronger. TNF-α and IL-8 are pivotal pro-inflammatory cytokines to activate and sustain NF-κB activity (Rasmussen et al., 2008; Bouwmeester et al., 2004). *P. distasonis* has been demonstrated to reduce the generation of TNF-α and IL-1β and inhibit NF-κB signal pathway, then ameliorating the inflammatory response in rat acute ischemic stroke model (Wei et al., 2024). The ETEC K88 infected IPEC-J2 cells assay in this study *in vitro* also showed similar results of decreased TNF-α, IL-1β, and IL-8 at protein or gene levels after *P. distasonis* treatment, consist with the previous report (Wei et al., 2024; Wang et al., 2016). We didn't go further to explore the role of decreased TNF-α, IL-1β or IL-8 after *P. distasonis* treatment on activation of NF-κB signal pathway or other inflammation associated signal pathway, because we focused on the aspect of *P. distasonis*-induced in the alterations of gut microbiota in ETEC K88 infected mice. *P. distasonis* and *E. coli* Nissle 1917 alleviated the reduction of occludin, claudin-1 and ZO-1 levels in ETEC K88 infected mice ileum tissues, suggesting that these two novel probiotics could maintain the ileal barrier integrity to prevent ETEC K88-caused permeability of pathogen or ions (McLamb et al., 2013). What's more, immunohistochemistry indicated that *P. distasonis* and *E. coli* Nissle 1917 also counteracted epithelial barrier dysfunctions in ETEC K88 infected IPEC-J2 cells through improving the tight junction disruptions and distribution.

ETEC infection-induced diarrhea is associated with gut dysbiosis in piglets or mice (Zhang et al., 2022; Yue et al., 2020; Wang et al., 2018; Zhang et al., 2025; Bin et al., 2018; Chen et al., 2025; Xu et al., 2024; Yang et al., 2021). The present study evaluated and compared the impact of *P. distasonis* and *E. coli* Nissle 1917 on the gut microbiome in ETEC K88-infected mice. We found that ETEC K88 infection significantly decreased the α-diversity of gut microbiota, consist with the previous research (Yue et al., 2020). *P. distasonis* and *E. coli* Nissle 1917 both increased the diversity of microbial community damaged by ETEC, and *E. coli* Nissle 1917 exerted stronger up-regulation capability than *P. distasonis*. The heat map indicated that an increased abundance of *Akkermansia, Escherichia–Shigella, Parabacteroids* and *Bacteroides*, a decreased abundance of the Lachnospiraceae_NK4A136_group, *Alistipes, Lachnoclostridium, Oscillibacter*, and *Lactobacillus* were associated with ETEC K88 infection. Whereas, the supplementation with *P. distasonis* and *E. coli* Nissle 1917 could reshape the gut microbiota composition and structure. We found that *P. distasonis* treatment could recover the abundance of the Lachnospiraceae_NK4A136_group during ETEC infection. As short chain fatty acids (SCFAs) producing-bacteria, the Lachnospiraceae NK4A136 group was identified as a signature of healthy gut bacterium in one Austrian dementia study (Stadlbauer et al., 2020). The LEfSe analysis showed significant increased *B. thetaiotaomicron* was associated with ETEC K88 infection, significant increased *P. distasonis* and *B. uniformis* were associated with *P. distasonis* treatment in ETEC K88 infected mice. *B. thetaiotaomicron* usually degrades complex polysaccharides into monosaccharides which could be readily utilized by *E. coli* and *Clostridium difficile* (Sonnenburg et al., 2005; Xu et al., 2003). In addition, *B. thetaiotaomicron* is reported to colonize at enterohemorrhagic *E. coli* (EHEC) attachment sites and exacerbate EHEC infection through modifying the metabolites (Curtis et al., 2014). *B. uniformis* has been demonstrated to alleviate inflammation by reducing the expression of acyl carrier proteins and utilized as micro-ecologic to treat antibiotic-associated diarrhea (Guo et al., 2021). Subsequently, *P. distasonis* treatment could modulate the abundance of *B. uniformis* to control the ETEC K88 induced diarrhea. *B. thetaiotaomicron* could serve as a biomarker of ETEC K88 infection in mice and *B. uniformis* functions as a biomarker of *P. distasonis* treatment in ETEC K88 infected mice.

## Conclusion

Altogether, *P. distasonis* and *E. coli* Nissle 1917 both exerted protective effects on mediating ETEC-induced diarrhea in a mouse model, with comparable efficacy. Our findings indicate that *P. distasonis* could down-regulate pro-inflammatory cytokines and up-regulate tight junction protein levels *in vitro* and *in vivo*. Moreover, *P. distasonis* could improve the perturbations in the gut microbiota induced by ETEC K88. The deficiency of beneficial bacteria such as the Lachnospiraceae_NK4A136_group and *B. uniformis* were improved by *P. distasonis* treatment. Collectively, the present study demonstrates that *P. distasonis* and *E. coli* Nissle 1917 qualify as novel probiotics with potential protective effects against ETEC-induced diarrhea.

## Data Availability

The data presented in this study are publicly available. The data can be found here: https://www.ncbi.nlm.nih.gov/, accession SRP626360 (PRJNA1333859).

## Supplementary material

Supplementary Tables S1, S2 Primer sequences for porcine and murine target genes.

Supplementary Figure S1 IPEC-J2 cell viability after *P. distasonis* and *E. coli* Nissle 1917 treatment.

Supplementary Figure S2 OTU rank-abundance curves for all samples.
