# Supplementary material for: Parabacteroides distasonis alleviates enterotoxigenic Escherichia coli-induced diarrhea in mice by mediating gut microbiota
Source: Front Microbiol. 2026 Feb 6;16:1716958. doi: 10.3389/fmicb.2025.1716958 (PMC12922235; doi:10.3389/fmicb.2025.1716958)
Supplement: Supplementary Tables S1, S2 — Primer sequences for porcine and murine target genes. [file Supplementary_file_1.docx]

Table S1 Primer sequences for porcine target genes

| Target genes | sequences | numbers of base pair |
| --- | --- | --- |
| TNF-α F  TNF-α R  IL-8 F  IL-8 R  IL-1β F  IL-1β R  β-actin F  β-actin R | TCCARATGGCAGAGTGGGTATG  AGCTGGTTGTCTTTCAGCTTCAC  ACAGCAGTARACARACARACARAG  GACCAGCACAGGARATGAG  GGCTACTGCCTTCCCTACC  CCTGATTGAACCCAGATTGG  TGCGGGACATCARAGGAGARAG  AGTTGARAGGTGGTCTCGTGG | 22  23  24  19  19  20  22  21 |

Table S2 Primer sequences for murine target genes

| zo-1 F  zo-1 R  Occludin F  Occludin R  Claudin-1 F  Claudin-1 R  TNF-α F  TNF-α R  IL-8 F  IL-8 R  CRP F  CRP R  β-actin F  β-actin R | AGCCTTGCAAAGCCAGCTCA  AGTGGCCTGGATGGGTTCATAG  AAGAGTTGACACTCCCATGGCATAC  ATCCACAGGCCAAGTTAATGGAAG  GCATGAAGTATATGAAGTGCTTGGA  CGATTCTATTGCCATACCATGCTG  AAACCACCAAGTGGAGGAGC  ACAAGGTACAACCCATCGGC  CAGTTTTGCCAAGGAGTGCTAA  AACTTCTCCACAACCCTCTGC  TTTCGCTAGCATGGAGAAGCTACTCTGG  GAACGAATTCTCAGGACCACAGCTGCG  AGGCCAACCAACCGCGAGAAG  GTCCAGGCGCATGATGG | 20  22  25  24  25  24  20  20  22  21  28  27  25  17 |
| --- | --- | --- |
