# Supplementary figures and images for: Parabacteroides distasonis alleviates enterotoxigenic Escherichia coli-induced diarrhea in mice by mediating gut microbiota
Source: Front Microbiol. 2026 Feb 6;16:1716958. doi: 10.3389/fmicb.2025.1716958 (PMC12922235; doi:10.3389/fmicb.2025.1716958)

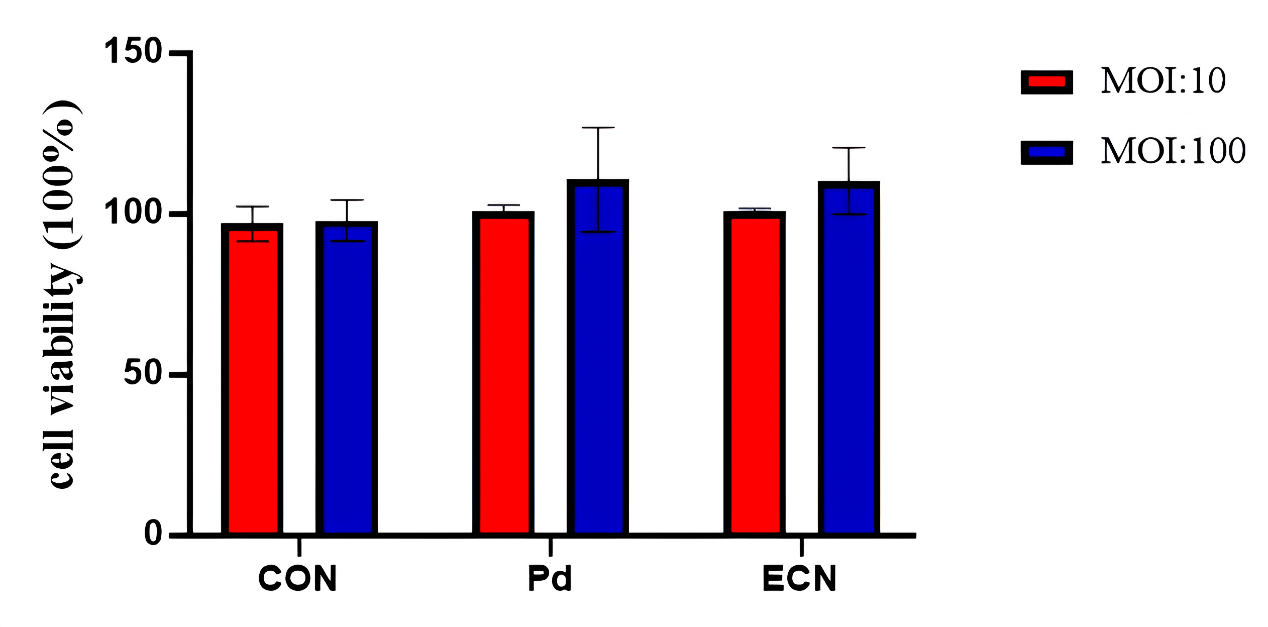


IPEC-J2 cell viability after *P. distasonis* and *E. coli* Nissle 1917 treatment.

Supplement: Supplementary Figure S1 — IPEC-J2 cell viability after P. distasonis and E. coli Nissle 1917 treatment. [file Supplementary_file_2.docx]

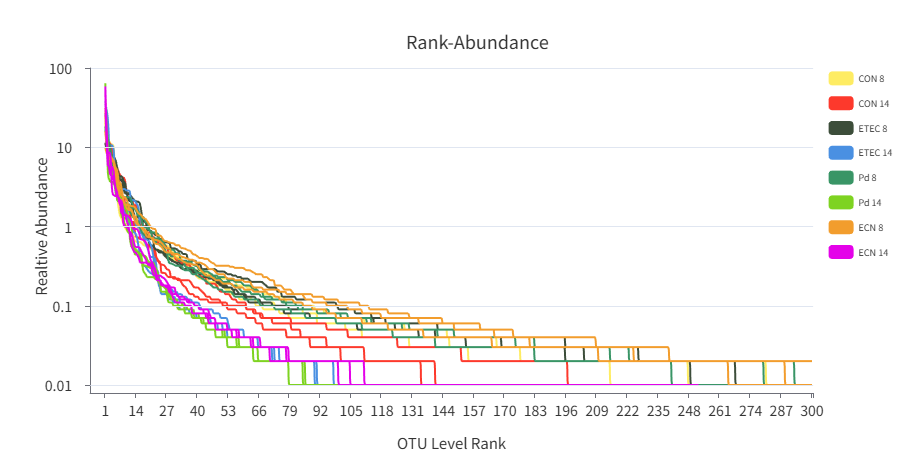
 OTU rank-abundance curves

Supplement: Supplementary Figure S2 — OTU rank-abundance curves for all samples. [file Supplementary_file_3.docx]
